# Lateral habenula neurons signal step-by-step changes of reward prediction

**DOI:** 10.1016/j.isci.2022.105440

**Published:** 2022-10-27

**Authors:** Hyunchan Lee, Okihide Hikosaka

**Affiliations:** 1 Laboratory of Sensorimotor Research, National Eye Institute, National Institutes of Health, Bethesda, MD 20892-4435, USA

**Keywords:** Biological sciences, Neuroscience, Behavioral neuroscience

## Abstract

In real life, multiple objects of different values are mixed in a variety of environments. To survive, animals need to find rewarding objects that may be located but hidden in particular contexts (e.g., environments) with bad objects that are unassociated with reward. Then, animals and humans pay attention to the enriched environment so that they can find the rewarding object vigorously. How can the brain initiate such behavior based on the context? We thus created a behavioral task for monkeys in which multiple contextual events (environment, action cue) sequentially occurred before objects appeared. We then studied the lateral habenula (LHb), which inhibit dopamine neurons (Matsumoto and Hikosaka, 2007). LHb neurons showed phasic responses in each event step-by-step across the sequential events, whose direction (excitation or inhibition) corresponded to the immediate change of the predicted value. Moreover, LHb neurons sequentially compared detailed prediction errors based on their significance in multiple contexts.

## Highlights

• Lateral habenula encoded errors in sequential events based on the latest prediction
• They discriminated multiple dimensions of contexts within each step sequentially
• The sequence of discrimination was based on the strength of value discrimination
• The greatest factor determined initial response classified into inhibition/excitation

## Graphical abstract

**Figure:**
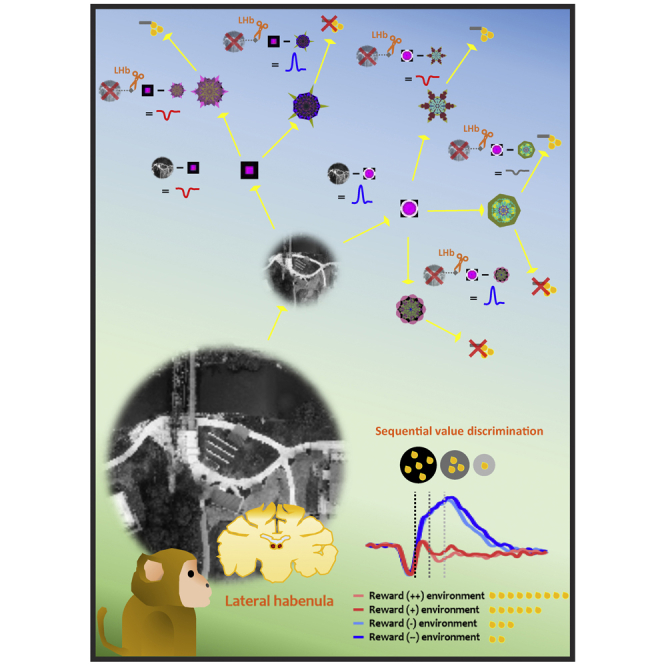

## Introduction

To get a reward, we often need to find an object that provides a reward (Carter and Tiffany, 1999; Ghazizadeh et al., 2016b; Nijs et al., 2010). This can be done by the basal ganglia (BG) based on the input from dopamine (DA) neurons (Kim and Hikosaka, 2015; Nakamura and Hikosaka, 2006).

Basic information carried by DA neurons is reward prediction error (RPE) which indicates the change of reward value: received value – predicted value (Eshel et al., 2015; Schultz et al., 1997). Then, DA neurons are retrospectively excited if RPE is positive or inhibited if RPE is negative. The increase of DA release would facilitate the synaptic plasticity of neurons in BG through D1 receptors in the direct pathway and D2 receptors in the indirect pathway (Calabresi et al., 2007; Hong and Hikosaka, 2011). Thereby, DA release would reinforce synaptic pathways that are consistent with experienced outcomes. Notably, this retrospective RPE response to the outcome of DA neurons would control the output of BG at the end of behavior, not the beginning of behavior. Then, goal-directed behavior would not be changed.

However, DA neurons are also enabled to prospectively change their activity successfully if particular objects appear consistently and selectively before the reward outcome: e.g., Excitation to object A, which precedes reward; Inhibition to object B, which precedes no reward (Lak et al., 2014; Matsumoto and Hikosaka, 2009b; Takikawa et al., 2004). We will call these activities ‘Anticipatory-RPE’. During this learning process, retrospective RPE activity (after reward) is replaced by Anticipatory-RPE activity (before reward; after object) within several trials (Takikawa et al., 2004). This temporal (step-by-step) change of activity in DA neurons is very important for the learning of goal-directed behavior. However, the underlying mechanism for this process is still unclear.

This may be provided by the change of inputs to DA neurons. One candidate is the lateral habenula (LHb) which inhibits DA neurons through rostromedial tegmental nucleus (RMTg) (Hong et al., 2011; Jhou et al., 2009). LHb is now thought to be one of the most critical brain areas that control emotion, and its dysfunction is related to depression, schizophrenia, and drug-induced psychosis (Hikosaka, 2010). Neurons in LHb are sensitive to the pre-reward events (Bromberg-Martin and Hikosaka, 2011), which may create Anticipatory-RPE responses in DA neurons (Schultz et al., 1993).

**Figure 1:**
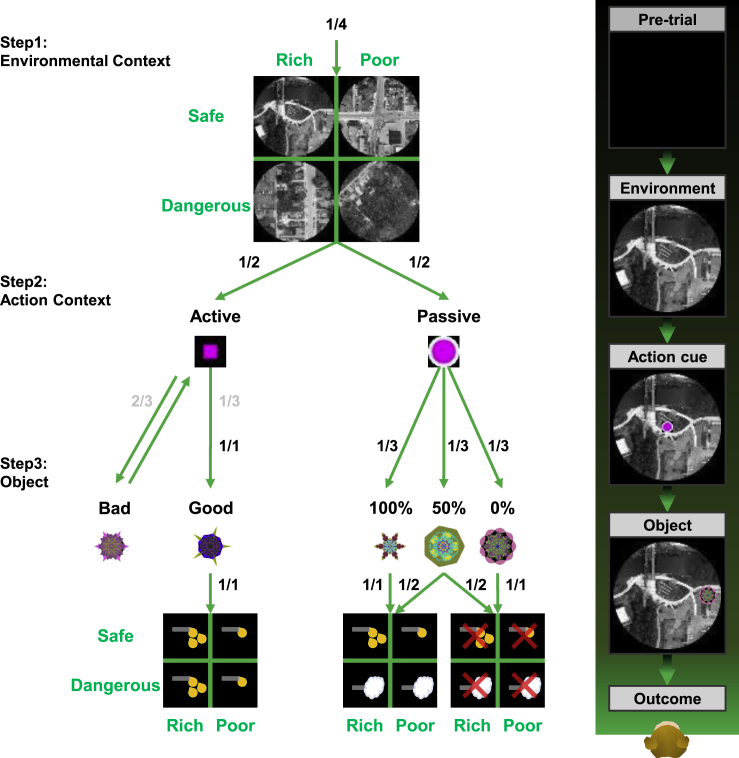
Multi-stage contingency task Sequence of events in task procedure. Environment, action cue, and object were presented on the screen in a row. Environments were assigned to one of four contexts (Rich-Safe, Poor-Safe, Rich-Dangerous, Poor-Dangerous) according to the experienced outcome types within each environment. Each environment contained two distinct objects for the active mode and three objects for the passive mode. After the action cue, a fractal object was presented at left or right position. In the active mode, monkeys requested to make a saccade to the good object and to avoid the bad object. In the passive mode, monkeys could freely observe the object. After the instrumental/natural viewing, monkeys received respective outcomes associated with the individual object. The objects of Rich-context were associated with high-valued reward, whereas the objects of Poor-context were associated with low-valued reward. In the passive mode, objects of Safe-context were associated with the same amount of reward in the active mode with different probabilities (100%, 50%, and 0%), but the objects of Dangerous-contexts were associated with airpuff (100%, 50%, and 0%).

**Figure 2:**
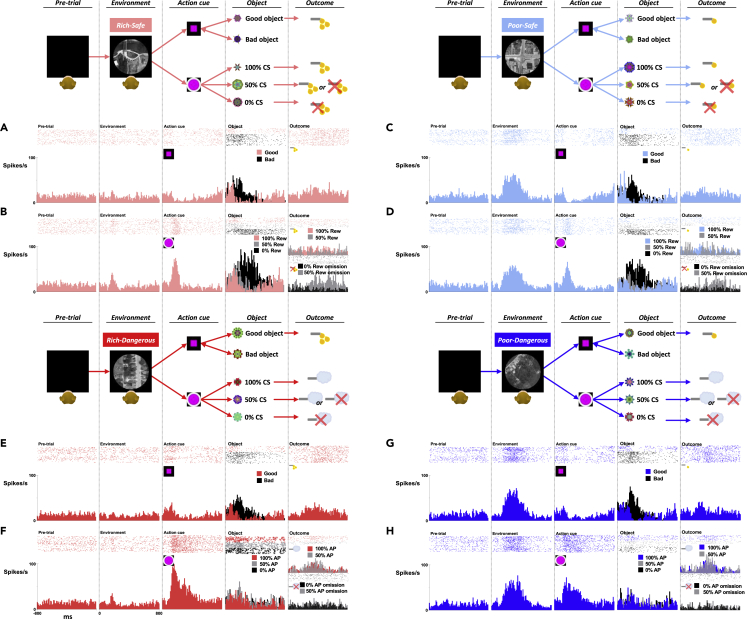
LHb activities at eachstep and each context (A, C, E, and G) Activities of an example LHb neuron at each step (Pre-trial, Environment, Action cue, Object, Outcome) during the active mode. (B, D, F, and H) Activities of the example LHb neuron during the passive mode.

We thus studied the activity of LHb neurons in monkeys by creating complex behavioral task (Figure 1) for two reasons. First, in real life, there are often many events before the reward outcome, which occur sequentially and in parallel. Second, many events are based on contexts (e.g., environment, action), each of which is not associated selectively with a particular outcome (unlike objects). As a whole, one particular event (object, environment, action, probability) does not predict a particular outcome. Instead, the outcome varies depending on the preceding and subsequent events. Indeed, LHb neurons responded to each of many events phasically, which encoded Anticipatory-RPE at each event selectively (Figures 2 and S1).

## Results

### Contingency task for inducing updated reward predictions

To test how LHb neurons are affected by informative context, we recorded LHb neurons of monkeys performing a multi-stage contingency task consisting of three sequential events. The three-event stages comprised an environment cue (Step 1), an action cue (Step 2), and a fractal object (Step 3), each of which progressively constrained the trial outcome through a set of hierarchical contingencies (Figure 1). Each successive event conveyed information to the monkey constraining the possible range of trial outcomes (large reward, small reward, airpuff punishment, or nothing), which in some conditions remained in doubt until the very end of the trial. Each trial began with the onset of the environment cue, which was a large (40 deg) circular image drawn from a 2x2 matrix of conditions determining two conditions, namely, reward volume (Rich versus Poor) and potential for punishment (Dangerous versus Safe). Next, an action cue appeared in the center of the screen that signaled either the passive mode (round fixation spot) or the active mode (square fixation spot).

In the passive mode, the remainder of the trial amounted to a Pavlovian task in which an object (fractal image) appeared and served as a conditioned stimulus (CS), indicating the probability (100%, 50%, or 0%) that an unconditioned stimulus (US) would occur. There were no action requirements for the monkeys. The US was the delivery of either an airpuff (in Dangerous-environments) or a juice reward (in Safe-environments). In the latter case, the juice reward was either large (600 μL) or small (200 μL) in Rich versus Poor environments, respectively.

In the active mode, the remainder of the trial was an instrumental saccade task in which either a good fractal object appeared (⅓ of active trials) and signaled the availability of a reward, or a bad object appeared (⅔ of active trials) and signaled no reward. The monkey could claim the rewards by making an eye movement to the good object and maintaining fixation for 500 ms. In all bad object trials, if the monkey fixated on a bad object, the trial was aborted and the same condition was repeated on the trial until a correct response was achieved (i.e., withholding fixation on the bad object). After rejecting the bad object, the active action cue re-appeared and the good object subsequently appeared. Thus, the monkey was allowed to make saccade to the good object and claim the reward as usual. As in the passive mode, reward volume was either 600 μL or 200 μL, depending on whether the environment context was Rich or Poor. Unlike the passive mode, the Dangerous versus Safe context was irrelevant, as the threat of airpuff did not apply in the active mode. Thus, monkeys experienced a larger amount of reward with no threats in the active mode than in the passive mode.

Indeed, there are various contexts in real life, including spatial, temporal, social, and cultural contexts (Maren et al., 2013), in which humans and animals perform reward-oriented behaviors. Depending on the context, they may be able to get a reward either actively or passively. Although the active action could be difficult, the subject can get the reward consistently by choosing good objects. In fact, in the present study, the monkeys were able to choose good objects and reject bad objects almost completely (error rate <2%) after several blocks of learning (>5 blocks). In the passive context, such learning is unnecessary, but sometimes the subject may not obtain a reward and face a risky outcome. These events actually occur in this task procedure (Figure 1). How then do LHb neurons respond to these environments that various contexts are mixed? Before this question, we studied the activity of LHb neurons in response to the free outcome: we presented reward or airpuff spontaneously without preceding event or context. The LHb neurons were inhibited by reward and excited by airpuff, as shown in the previous study (Matsumoto and Hikosaka, 2009a) (Figure S2A).

Crucially, the sequential and hierarchical arrangement of task components allowed the subjects to proceed from a state of greater to lesser uncertainty as the trial progressed. Each condition was specified by a combination of environment cue, action cue, and fractal object, thus giving rise to a quantifiable reward prediction (RP) that updated with the onset of each trial event. Supplementary Tables 1-2 of the previous study (Lee and Hikosaka, 2022) summarize how reward (in microliters of juice) and punishment predictions (in milliseconds of airpuff) were determined according to all combinations of task conditions. Because all the stimuli and outcomes appeared pseudorandomly, we could compute the theoretical RP at each step (Figure S1). In the following sections, we examine in turn how LHb neurons were affected by each of these events and their corresponding updates to Anticipatory-RPE.

### Lateral habenula sensitivity to the firststep: Environment

**Figure 3:**
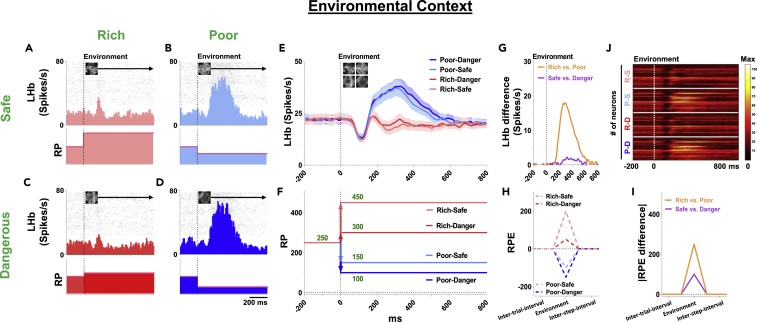
LHb responses to environmental context (A–D) Top, responses of an example LHb neuron to environments. Rasters and histograms (10 ms bins) are aligned by environment onset. Bottom, theoretical RP to the environment. (E) Population average firing rates of LHb neurons. (F) Theoretical RP to the environmental scene (Rich-Safe, 450; Rich-Dangerous, 300; Poor-Safe, 150; Poor-Dangerous, 100). (G) The differences in LHb response between the Rich-versus Poor-contexts (orange) and Safe-versus Dangerous-contexts (violet). (H) The difference in RP (RPE) between before and after the environment onset. (I) The differences of theoretical RPE between the Rich-versus Poor-contexts (orange) and Safe-versus Dangerous-contexts (violet). (J) The activity of individual LHb neurons (n = 33) before and after the start of environment onset. Each condition (R-S, Rich-Safe; P-S, Poor-Safe; R-D, Rich-Dangerous; P-D, Poor-Dangerous) is aligned in the same step.

We examined LHb activity in Step 1: Environment (Figure 3), which is shown by one LHb neuron (Figures 3A–3D) and the average activity of 33 LHb neurons (Figure 3E). They discriminated two kinds of environmental context sequentially, as shown below (A) and (B).

(A) first effect of environment cue (Poor versus Rich context):

After any of the environments appeared, LHb neurons were initially inhibited during 50–150 ms after its onset (Figures 3E and 3J). Then, the LHb neurons started changing their activity: excited by Poor-environments and inhibited by Rich-environments. This occurred in all LHb neurons in general (Figure S3B).

To study how LHb neurons respond to the environment, we compared the LHb neuronal responses with RP (Figure S1). The theoretical values of RP were calculated by the amount and probabilities of outcome after each step. On the basis of amount (Rich, 600 μL; Poor, 200 μL) and probabilities (active, 100%; passive, 50% in Safe and 0% in Dangerous) of reward in upcoming processes, each environment contextually updated different RP (Updated-RP) (Figure 3F), as indicated below.

  Updated-RP for environment (Figures 3F and S1):
    Rich-Safe: 600 μL × (100% in active +50% in passive)/2 = 450 μL
    Rich-Dangerous: 600 μL × (100% in active +0% in passive)/2 = 300 μL
    Poor-Safe: 200 μL × (100% in active +50% in passive)/2 = 150 μL
    Poor-Dangerous: 200 μL × (100% in active +0% in passive)/2 = 100 μL

Each of the Updated-RP was different from the predicted-value (Previous-RP, (450 + 300 + 150 + 100)/4 = 250 μL) before the environment onset. We then computed the Anticipatory-RPE elicited by each environment. The Anticipatory-RPE is the discrepancy of RP between two sequential events, current event (Updated-RP) and previous event (Previous-RP).

Anticipatory-RPE = Updated-RP – Previous-RP

  Anticipatory-RPE (Figures 3H and S1):
    Rich-Safe: 450 - 250 = +200 μL
    Rich-Dangerous: 300 - 250 = +50 μL
    Poor-Safe: 150 - 250 = −100 μL
    Poor-Dangerous: 100 - 250 = −150 μL

These Anticipatory-RPE were negatively correlated with LHb response to each context: Inhibition in Rich-environments, Excitation in Poor-environments. In summary, the first discriminations in LHb neuronal responses were classified into two groups (Inhibition versus Excitation) depending on the negative/positive discrimination of Anticipatory-RPE.

(B) second effect of environment cue (Dangerous versus Safe context):

Somewhat later (>200 ms), LHb neurons became slightly more active in Dangerous-environment than Safe-environment (Figures 3E and S3B). This was related to the difference of Anticipatory-RPE between Safe-environments and Dangerous-environments (Figures 3H and S1):

  Rich-Safe: +200 > Rich-Dangerous: +50
  Poor-Safe: −100 > Poor-Dangerous: −150

In these two steps of environmental effect, the activity of LHb neurons was negatively correlated with Anticipatory-RPE based on 2-dimensions of context (Figures 3E and 3F): (A) Poor > Rich, (B) Dangerous > Safe. Importantly, these 2-dimensional contexts modulated LHb neuronal activity sequentially: Poor versus Rich, then Dangerous versus Safe.

Why did these contexts change LHb neuronal activity separately and sequentially (Figure 3G)? This may be based on the strength of value discrimination in each context (Figure 3I):

1) Value discrimination in Rich-Poor context: 250
  Average value in Rich: +125 (Rich-Safe: +200, Rich-Dangerous: +50)
  Average value in Poor: −125 (Poor-Safe: −100, Poor-Dangerous: −150)
2) Value discrimination in Safe-Dangerous context: 100
  Average value in Safe: +50 (Rich-Safe: +200, Poor-Safe: −100)
  Average value in Dangerous: −50 (Rich-Dangerous: +50, Poor-Dangerous: −150)

Then, the discrimination of LHb neuronal activity occurred earlier when the discrimination of the predictive value was larger: Rich-Poor (250) followed by Safe-Dangerous (100).

### Lateral habenula sensitivity to the second step: Action cue

The next step of this task was action cue (Figure 1). The action cue (Step 2) was shown by a small symbol (magenta square or circle) at the center of the environment and instructed what the upcoming action mode (active or passive) is. The monkeys then performed one of two modes depending on the cue. Notably, each action cue did not predict a particular reward outcome, which means that these action cues acted as another context.

**Figure 4:**
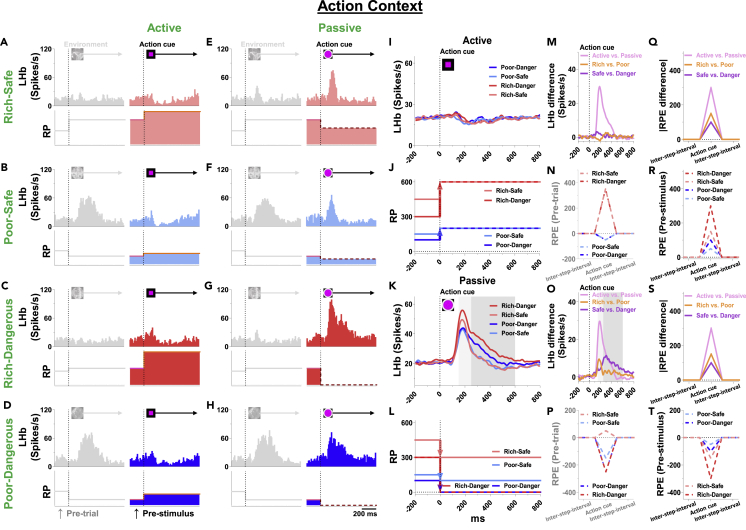
LHb responses to action context (A–H) Top, responses of an example LHb neuron to active- and passive-action cues. The neuronal activities of LHb were inhibited by the active-action cue (A–D), whereas those were greatly excited by the passive-action cue (E–H). Bottom, RP to action cue. RP was increased by the active-action cue, whereas the passive-action cue decreased it in all contexts. The gray histograms and graphs indicate LHb activities and theoretical RP in the preceding step. (I) Population average firing rates of LHb neurons aligned to active-action cue onset. (J) Theoretical RP to active-action cue (Rich-Safe, 600; Poor-Safe, 200; Rich-Dangerous, 600; Poor-Dangerous, 200). (K) Population average firing rates of LHb neurons aligned to passive-action cue onset. Shaded areas indicate early (light gray, 150-250 ms) and late phases (dark gray, 250-600 ms), representing distinct secondary and tertiary contextual value discrimination. (L) Theoretical RP to passive-action cue (Rich-Safe, 300; Poor-Safe, 100; Rich-Dangerous, 0; Poor-Dangerous, 0). (M and O) The differences in LHb response between the contexts (magenta, Active versus Passive; orange, Rich versus Poor; violet, Safe versus Dangerous). (N and P) Theoretical Pre-trial-based RPE, the difference in RP between before environment onset and after the action cue onset. (R and T) Theoretical Pre-stimulus-based RPE, the difference in RP between before and after the action cue onset. (Q and S) The differences of theoretical RPE (Pre-stimulus-based) between Active-versus Passive-action cue (magenta) Rich-versus Poor-contexts (orange), and Safe-versus Dangerous-contexts (violet).

LHb neurons then discriminated 3-dimensions of context sequentially, especially in passive mode (Figure 4K), as shown in below (A) and (B).

(A) First effect of action cue (Active versus Passive context):

In the active mode, one of two fractal objects (Figure 1, good or bad) appeared in random sequence at left or right position. To get a reward, the monkey needed to choose the good object by making a saccade to it and watching it (i.e., keeping gaze >500 ms). To reach this goal, the monkey also needed to reject the bad object in either of two ways: 1) No saccade to it, 2) Going away from it within 500 ms after making a saccade to it.

When the monkey failed appropriate action occasionally (<2%), the trial was stopped, and the same trial was repeated until it was succeeded. Thus, the monkey finally obtained reward in the 100% trials of active mode. Therefore, the active-action cue induced high Updated-RP (Figure 4J).

  Updated-RP for active-action cue (Figures 4J and S1):
    Rich-Safe: 600 μL × 100% = 600 μL
    Rich-Dangerous: 600 μL × 100% = 600 μL
    Poor-Safe: 200 μL × 100% = 200 μL
    Poor-Dangerous: 200 μL × 100% = 200 μL

In the passive mode, one of three fractal objects (Figure 1) appeared at left or right position. Each of them was followed by a particular outcome, regardless of the monkey’s behavior: 100%, 50%, 0% reward in Safe-contexts or 100%, 50%, 0% airpuff in Dangerous-contexts. In contrast to the active mode, there was no way for the monkey to choose or reject them. The outcome was decided by each object, not the monkey’s behavior. Therefore, the passive-action cue induced lower Updated-RP (Figure 4L) than the active-action cue (Figure 4J).

  Updated-RP for passive-action cue (Figures 4L and S1):
    Rich-Safe: 600 μL × (100% + 50% + 0%CS)/3 = 300 μL
    Rich-Dangerous: 600 μL × 0% = 0 μL
    Poor-Safe: 200 μL × (100% + 50% + 0%CS)/3 = 100 μL
    Poor-Dangerous: 100 μL × 0% = 0 μL

First of all, they were inhibited by active-action cues (Figures 4A–4D and 4I) and excited by passive-action cues (Figures 4E–4H and 4K). These data suggest that the LHb responses would be negatively correlated with the Anticipatory-RPE, similarly to the environment responses (Figure 3). But this raised an important question, as shown below.

For the environment responses (Figure 3), Anticipatory-RPE was defined as the change of RP between before and after the current event, as described above.

  Environment cue:

Anticipatory-RPE = Updated-RP (= Environment) – Previous-RP (= Pre-trial)

For the action cue response, there were two steps before the current event. Is Previous-RP based on Pre-trial event (= Pre-trial) or Pre-stimulus event (= Environment)?

  Action cue:
    1) Anticipatory-RPE = Updated-RP (= Action) – Previous-RP (= Pre-trial)
    2) Anticipatory-RPE = Updated-RP (= Action) – Previous-RP (= Pre-stimulus)

We then examined which mechanism can predict the actual activity of LHb neurons.

1) Pre-trial-based Anticipatory-RPE
  Active-action cue (Figure 4N):
    Rich-Safe: 600 - 250 = +350 μL
    Rich-Dangerous: 600 - 250 = +350 μL
    Poor-Safe: 200 - 250 = −50 μL∗
    Poor-Dangerous: 200 - 250 = −50 μL∗
  Passive-action cue (Figure 4P):
    Rich-Safe: 300 - 250 = +50 μL∗
    Rich-Dangerous: 0 - 250 = −250 μL
    Poor-Safe: 100 - 250 = −150 μL
    Poor-Dangerous: 0 - 250 = −250 μL

First, these data (based on Pre-trial event) were not consistently correlated with the LHb responses because they were inhibited by all active-action cues (Figure 4I) and excited by all passive-action cues (Figure 4K). The opposite data are indicated by asterisks (∗).

2) Pre-stimulus-based Anticipatory-RPE
  Active-action cue (Figure 4R):
    Rich-Safe: 600 - 450 = +150 μL
    Rich-Dangerous: 600 - 300 = +300 μL
    Poor-Safe: 200 - 150 = +50 μL
    Poor-Dangerous: 200 - 100 = +100 μL
  Passive-action cue (Figure 4T):
    Rich-Safe: 300 - 450 = −150 μL
    Rich-Dangerous: 0 - 300 = −300 μL
    Poor-Safe: 100 - 150 = −50 μL
    Poor-Dangerous: 0 - 100 = −100 μL

On the other hand, all of these data (based on Pre-stimulus event) are consistent with the LHb responses: Inhibited by all active-action cues (Figure 4I) and excited by all passive-action cues (Figure 4K). These data suggest that LHb neurons predict an immediate change of RP.

(B) second and third effects of action cue (Rich versus Poor, Dangerous versus Safe context):

In addition to the difference between active- and passive-action cues, LHb neurons also discriminated other reward contexts, which were clearer in response to passive-action cue (Figures 4K, 4O, 4S, and S4D), compared with active-action cue (Figures 4I, 4M, 4Q, and S4C). During passive-action cues, the discrimination occurred in two additional sequences (Figures 4K and S4D):

  1) Rich > Poor (150-250 ms)
  2) Dangerous > Safe (250-600 ms)

The LHb neurons changed their activity based on 3-dimensions of contexts sequentially: 1. Passive versus Active, 2. Rich versus Poor (Figures 4K and 4O, light gray), 3. Dangerous versus Safe (Figures 4K and 4O, dark gray). These sequential context effects are consistent with the strength of value discrimination in each context (RS, Rich-Safe; RD, Rich-Dangerous; PS, Poor-Safe; PD, Poor-Dangerous) (Figure 4S):

  1) Value discrimination in Active-Passive context: 300
    Average value in Active: +150 (RS: +150, RD: +300, PS: +50, PD: +100)
    Average value in Passive: −150 (RS: −150, RD: −300, PS: −50, PD: −100)
  2) Value discrimination in Poor-Rich context (in Passive context): 150
    Average value in Poor: −75 (PS: −50, PD: −100)
    Average value in Rich: −225 (RS: −150, RD: −300)
  3) Value discrimination in Safe-Dangerous context (in Passive context): 100
    Average value in Safe: −100 (RS: −150, PS: −50)
    Average value in Dangerous: −200 (RD: −300, PD: −100)

As shown in Figures 3 and 4, LHb neurons responded to multiple contexts (Rich-Poor, Safe-Dangerous, or Active-Passive) sequentially at each cue (Environment, Action). Importantly, the sequence was consistently based on the strength of value discrimination in each context: Earlier if the value discrimination is larger.

### Lateral habenula sensitivity to the finalstep: Active target object

**Figure 5:**
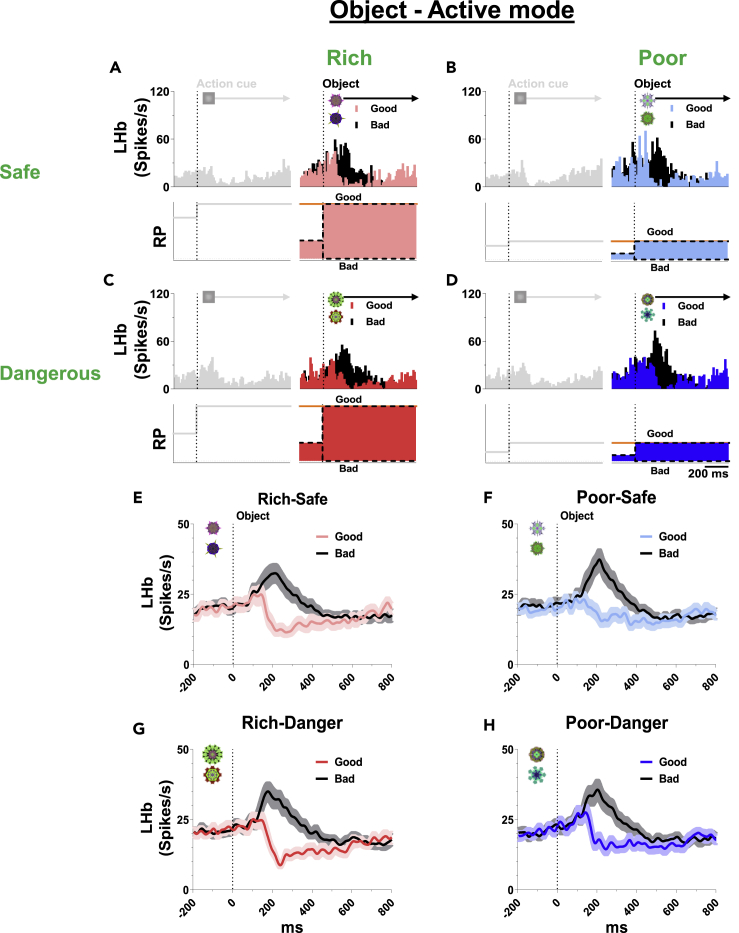
LHb responses to objects in active mode (A–D) Top, responses of an example LHb neuron to good and bad objects in active mode. LHb was inhibited by the good objects and excited by the bad objects. Bottom, theoretical RP based on environment (orange) and appearance-rate (33.3%, black dotted) of the good object. (E–H) Population average firing rates of LHb neurons in each context.

**Figure 6:**
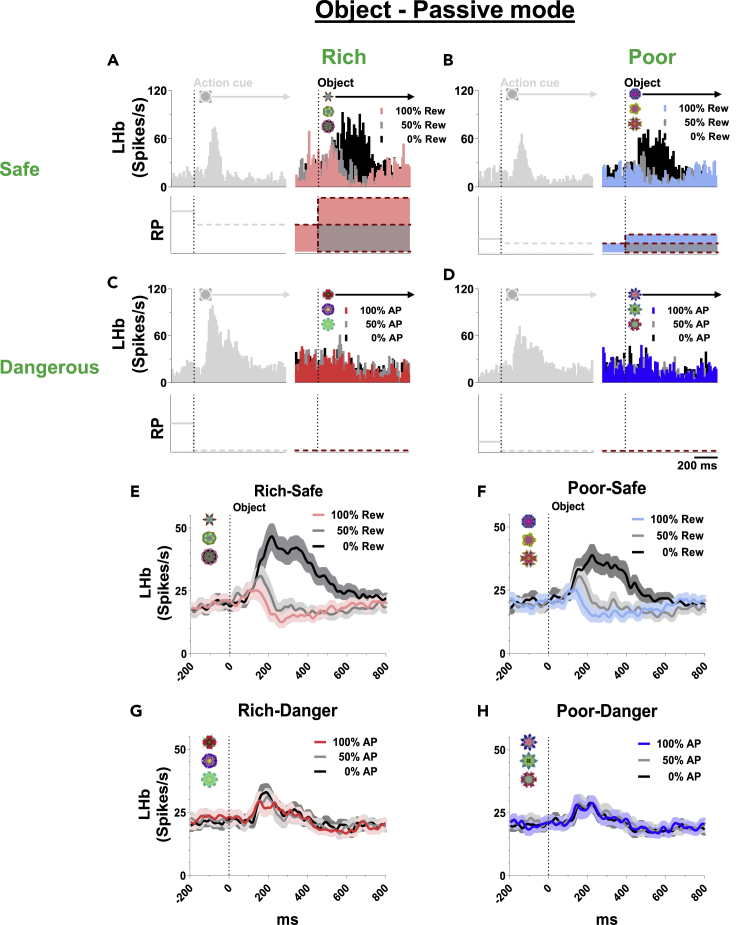
LHb responses to objects in passive mode (A–D) Top, responses of an example LHb neuron to objects in passive mode. Bottom, theoretical RP to object in passive mode. (E–F) Population average firing rates of LHb neurons in Safe-contexts. LHb was inhibited by 100%-reward CS and excited by 0%-reward CS in Safe-contexts. (G–H) Population average firing rates in Dangerous-contexts. LHb was slightly excited by airpuff CS in Dangerous-contexts. The probabilistic discrepancy was not observed among airpuff CS in Dangerous-contexts.

After the second step (action cue), the final step started and the outcome was delivered by a target object based on the monkey’s instrumental behavior (active) or regardless of the monkey’s behavior (passive) (Figure 1). We then studied the responses of LHb neurons to these objects (Figures 5 and 6).

In the active mode, LHb neurons were inhibited by good objects and excited by bad objects in all environments (Figure 5). Because the monkey had already learned to choose good object and reject bad object and the subject could repeat the same trial until it gets a reward, the Upcoming-RP of the good object corresponds to the reward amount of each context (bad object = 0 μL).

Updated-RP for good object (Figures S1A–S1D):
  Rich-Safe: 600 μL
  Rich-Dangerous: 600 μL
  Poor-Safe: 200 μL
  Poor-Dangerous: 200 μL

Then, this raises a question that we checked before: Are the responses of LHb neurons correlated with 1) Pre-trial event (= Pre-trial) or 2) Pre-stimulus event (= Active-action cue)?

1) Anticipatory-RPE = Updated-RP (= Object) – Previous-RP (= Pre-trial)
2) Anticipatory-RPE = Updated-RP (= Object) – Previous-RP (= Pre-stimulus)

First, we examined Anticipatory-RPE for good and bad objects based on Pre-trial event. As shown above, the average Previous-RP was 250 ul (Figure S1).

1) Pre-trial-based Anticipatory-RPE (Figures S1A–S1D)
  Pre-trial-based Anticipatory-RPE for good object:
    Rich-Safe: 600-250 = +350 μL
    Rich-Dangerous: 600-250 = +350 μL
    Poor-Safe: 200-250 = −50μL∗
    Poor-Dangerous: 200-250 = −50μL∗
  Pre-trial-based Anticipatory-RPE for bad object:
    Rich-Safe: 0-250 = −250 μL
    Rich-Dangerous: 0-250 = −250 μL
    Poor-Safe: 0-250 = −250 μL
    Poor-Dangerous: 0-250 = −250 μL

These PRE data were not correlated with some LHb responses which were inhibited by good objects in Poor-Safe and Poor-Dangerous contexts (Figures 5F and 5H), shown by asterisks (∗).

On the other hand, during the Pre-stimulus event, this active-action context had two subcontexts (good object and bad object). Because good and bad objects appeared randomly, the subject needed to change its action. Here, the bad object was twice as likely to appear as the good object: Good object 33.3%, Bad object 66.7%. According to this condition, the Previous-RP was 33.3% of the Updated-RP for good object, which is shown below and in Figures 5A–5D (black dotted line) and Figures S1A–S1D (red dotted line).

  Pre-stimulus-based Previous-RP (Figures S1A–S1D):
    Rich-Safe: 600 μL × 33.3% = 200 μL
    Rich-Dangerous: 600 μL × 33.3% = 200 μL
    Poor-Safe: 200 μL × 33.3% = 67 μL
    Poor-Dangerous: 200 μL × 33.3% = 67 μL

Then, Anticipatory-RPE was computed for good object and bad object as below based on Pre-stimulus event (i.e., immediately before the appearance of good or bad object).

2) Pre-stimulus-based Anticipatory-RPE (Figures S1A–S1D)
  Anticipatory-RPE for good object:
    Rich-Safe: 600 - 200 = +400 μL
    Rich-Dangerous: 600 - 200 = +400 μL
    Poor-Safe: 200 - 67 = +133 μL
    Poor-Dangerous: 200 - 67 = +133 μL
  Anticipatory-RPE for bad object:
    Rich-Safe: 0 - 200 = −200 μL
    Rich-Dangerous: 0 - 200 = −200 μL
    Poor-Safe: 0 - 67 = −67 μL
    Poor-Dangerous: 0 - 67 = −67 μL

These computational data suggest that LHb neurons would be inhibited by good objects and excited by bad objects in all contexts, which was consistent with the actual data (Figure 5). This confirms the data in previous events (Environment, Action cue): LHb neurons responded to each object as Anticipatory-RPE which was based on Pre-stimulus value, not Pre-trial value. However, the difference between Rich- and Poor-contexts (based on the computational data, shown above) was not very clear in LHb neuron activity (Figure S5).

### Lateral habenula sensitivity to the final step: Passive target object

After passive-action cue (Figure 1), one of three objects (CS) appeared that led to a particular outcome regardless of the monkey’s behavior, unlike the active mode. The outcome (US) could be predicted by the environment (Rich-Safe, 600 μL reward; Poor-Safe, 200 μL reward; Rich-Dangerous, 100 ms airpuff; Poor-Dangerous: 100 ms airpuff), but the probability of the outcome was different among the three objects (100, 50, 0% object). Hence, each object predicted different Updated-RP (Figure S1).

Updated-RP (Figures S1E–S1H):
  Rich-Safe: 600 (100% object), 300 (50% object), 0 (0% object) μl
  Poor-Safe: 200 (100% object), 100 (50% object), 0 (0% object) μl
  Rich-Dangerous: 0 (100, 50, 0% object) μl
  Poor-Dangerous: 0 (100, 50, 0% object) μl

Figure 6 shows that LHb neurons were inhibited by 100%-reward object and excited by 0%-reward object in the Safe-contexts (Rich-Safe and Poor-Safe). To the 50%-reward object, LHb showed intermediate response. How then did LHb neurons encode Anticipatory-RPE at this final step? As we described in other steps, there were different types of Previous-RP in 1) pre-trial state and 2) pre-stimulus state, which could trigger Anticipatory-RPE. We thus computed Anticipatory-RPE for each object in the two Safe-contexts where LHb responses were variable. Pre-trial state was immediately before the appearance of environment (i.e., 250 μL, Figures S1E and S1F).

1) Pre-trial-based Anticipatory-RPE (Figures S1E and S1F)
  Rich-Safe context:
    100% object: 600 - 250 = +350 μL
    50% object: 300 - 250 = +50 μL
    0% object: 0 - 250 = −250 μL
  Poor-Safe context:
    100% object: 200 - 250 = −50 μL∗
    50% object: 100 - 250 = −150 μL
    0% object: 0 - 250 = −250 μL

This pre-trial-based Anticipatory-RPE predicts the excitation of LHb neurons in response to the 100% object in Poor-Safe context (∗), but LHb neurons were inhibited (Figure 6F). Again, this pre-trial-based Anticipatory-RPE was not appropriate to elucidate the negative correlation with LHb responses.

Next, there was another Previous-RP: Pre-stimulus state. Unlike Pre-trial state, the Previous-RP was variable between the two environments (Rich-Safe: 300, Poor-Safe: 100) (Figures S1E and S1F). Thus, the Anticipatory-RPE was respectively computed in each environment for the three objects, as shown below.

2) Pre-stimulus-based Anticipatory-RPE (Figures S1E and S1F)
  Rich-Safe context:
    100% object: 600 - 300 = +300 μL
    50% object: 300 - 300 = 0 μL
    0% object: 0 - 300 = −300 μL
  Poor-Safe context:
    100% object: 200 - 100 = +100 μL
    50% object: 100 - 100 = 0 μL
    0% object: 0 - 100 = −100 μL

This pre-stimulus based Anticipatory-RPE was negatively correlated with the LHb responses (Figures 6E, 6F, S6A, and S6B). The excitatory response to 0% object was stronger when the Anticipatory-RPE was more negative:−300 μL (Rich-Safe context) <−100 μL (Poor-Safe context) (Figure S7).

In summary, these data suggest that the phasic response of LHb neurons to each event (i.e., environment, action, object) is negatively correlated with the immediate change of RP at each event. The LHb neurons responded to the Anticipatory-RPE based on the latest pre-stimulus event. Thereby, the LHb neurons flexibly updated reward information at each step. In addition, these effects were particularly early and discriminative based on the strength of the value discrimination.

## Discussion

### LHb signals step-by-step changes of reward prediction

We found that LHb neurons are selectively sensitive to the change of value in each event of sequential behavior (i.e., Anticipatory-RPE) using phasic response. There were many sequential and parallel steps in this task before the outcome appeared. These steps were different based on multiple features (Figure 1): 1) Environment (Rich-Poor, Safe-Dangerous), 2) Action (Active-Passive), 3) Object (Good-Bad). The LHb neurons encoded all of these steps and features by changing their activity at each step to phasic excitation or inhibition. In other words, LHb neurons indicate how the current step is good (i.e., inhibition) or bad (i.e., excitation) along the whole sequence of events. This is critical to learn and modulate sequential behaviors in real life because the goal would often be reached after several events and behavioral responses. Many events occur step-by-step before the outcome, which together provide information about the final outcome. Recent studies have reported that the activities of DA neurons, a major target of LHb, represent moment-by-moment changes in the expected value in a decision-making task involving multiple task events (Kim et al., 2020; Tsutsui-Kimura et al., 2020; Wang et al., 2021). As animal behaviors are coordinated by multiple factors of past experiences that are able to appear in different temporal sequences, these step-by-step and moment-by-moment changes in LHb and DA will be crucial to modulating their sequential movements. Especially, it would be more important for animal behaviors in real reward environments beyond experimental conditions which often have higher volatility and complexity. Notably, a previous study reported that Parkinson's disease patients who are presumed to have dysfunction of the DA system exhibited disturbance of sequential movements (Benecke et al., 1987). These previous studies and present findings could support the evidence that the LHb-DA system might be critical for the multiple sequential early processes that way before a goal.

On the other hand, in this task consists of multiple sequential early processes ahead (Step 1: Environment, Step 2: Action cue, Step 3: Object), LHb neurons showed weaker response to punishment objects (Figures 6G and 6H) compared with reward objects (Figures 6E and 6F). In the previous study tested the Pavlovian task with a smaller sequence ahead of the objects (Step 1: Timing cue, Step 2: Object), LHb neurons sensitively discriminated 100%, 50%, and 0% airpuff probabilities of the objects (Matsumoto and Hikosaka, 2009a). We thus hypothesize that the aversiveness uncertainty could be an important source of triggering the aversive signaling of LHb neurons. Uncertainty is especially a fundamental source of LHb response in reward context as if the LHb neurons show an absence of their response when the delivery (Figures S2B and S2C) or omission (Figures S2D and S2E) of the outcome was completely predicted. Moreover, the LHb responses to the 50% reward object, which could be boosted by the reward uncertainty, have been observed as a response closer to 100% reward object than 0% reward object (Figures 6E and 6F). Along the same lines, LHb showed a stronger response to uncued free reward outcome than cued reward outcome associated with the 100% or 50% object (Figures S2B and S2C). This phenomenon is consistently observed in the aversive response of LHb neurons that showed a stronger response to uncued free airpuff than cued airpuff associated with the 100% or 50% object (Figures S2F and S2G). This result suggests that the aversive response of LHb could be sensitively desensitized by the expectedness of predictive cues and recent rodent studies have reported supportive evidence for this hypothesis (Jhou, 2021). Thomas C. Jhou and colleagues found that inactivation of LHb abolished the response of RMTg to the surprising uncued shock but not to the predictable cued shock (Li et al., 2019). In contrast with the conventional synaptic response, LHb neurons have distinct electrophysiological property which triggers great discharges of action potentials by activation of inhibitory synapses (Chang and Kim, 2004). Based on this mechanism, we assume that the sequential response of LHb in each step would tightly interact with each other. For example, the excitatory response to the punishment object in the present results (Figures 6G and 6H) could be suppressed and was unable to discriminate the airpuff probabilities because the preceding cue (passive action cue) induced long-lasting excitation and absence of inhibition of LHb activities in the previous step (Figure 4K).

### Sequential LHb response to discriminate multiple contexts

In this task, it was impossible to predict the outcome except for the final step (Figure 1), because RP changed at each step of these contexts and objects (Figure S1). This is, in fact, similar to what we (or animals) experience in real life (Kahneman and Tversky, 1979; Koszegi and Rabin, 2006). Reward outcome is often predictable by an object. However, each object may cause different outcomes in real life depending on various contexts (e.g., environment). How then can we predict reward and behave properly in the context? The context consists of multiple heterogeneous contextual elements. Thereby, the value of a goal often can’t be foreseen completely with only one event or element. Thus, it would be important to evaluate and integrate the values of multiple events or elements in the context. Then, the value of the context needs to be precisely compared with that in other contexts.

We found that LHb neurons were sensitive to the current and preceding contexts at each step to encode Anticipatory-RPE correctly. Of interest, their activity occurred separately and sequentially for different sets of Anticipatory-RPE based on different contexts. For example, in response to the Passive-action cue, there were three steps of excitation: 1) Passive > Active, 2) Rich > Poor, 3) Dangerous > Safe (Figures 4K, 4O, and S4D). This sequence was correlated with the strength of Anticipatory-RPE in each context: earlier if the negative strength was larger (Figure 4S; Passive-Active: 300 > Rich-Poor: 150 > Dangerous-Safe: 100).

Such sequential activities also occurred in response to other stimuli: Environment (Figures 3E and S3B). In response to the environments, there were two steps of excitation: 1) Poor > Rich, 2) Dangerous > Safe (Figure 3G). This sequence was also correlated with the strength of Anticipatory-RPE in each context (Figure 3I; Poor-Rich: 250 > Dangerous-Safe: 100). These data indicate that LHb neurons are sensitive to any change in RP across cue steps (Environment, Action, Object) as well as within each step (Rich-Poor, Safe-Dangerous, Active-Passive, Good-Bad objects). Then, any brain areas receiving inputs from LHb would be aware of step-by-step changes of RP (i.e., Anticipatory-RPE), which is important to learn and control goal-directed behaviors in multiple contexts (Ghazizadeh et al., 2016a). Moreover, RPE response in DA neurons is reported that be integrated by rewards of multiple dimensions (Lak et al., 2014). DA system has been globally thought to play a critical role in human motor function (Duke et al., 2007). A recent study reported that DA neurons modulate the initiation of action (da Silva et al., 2018). Especially to initiate an action efficiently, the LHb-DA system could play a crucial role in learning and monitoring the significance of the event based on multiple contexts.

Further studies examining the distinct reward information carried by LHb-projecting neurons from broad brain regions can provide an important hint on how complex and multiple reward values are analyzed and calculated in neuronal mechanisms. For example, tonically active interneurons (TANs) in caudate encode whether the context requires reward discrimination or not (Shimo and Hikosaka, 2001). This could be one of the profound sources of Previous-RP contextually generated and monitoring the significance of an upcoming event.

### Learning of Anticipatory-RPE

How can LHb neurons be sensitive to all early information along with the step-by-step processes? To control sequential processes, LHb neurons may get variable information from many brain areas. Indeed, LHb receives various inputs from broad brain regions that each area encodes one or a few different contexts and could not control all the information alone (Hu et al., 2020). Especially, LHb receives RPE signals through the multiple inputs from basal ganglia that are a pivotal source of learning. For example, LHb receives strong projection from the internal segment of the globus pallidus (GPi) and ventral pallidum (Hong and Hikosaka, 2013). Previous studies found that a group of neurons in the dorsal striatum (caudate and putamen) (McClure et al., 2003) and GPi (Hong and Hikosaka, 2008) represent RPE responses. For more specific, the patchy regions (striosome) of the dorsal striatum are connected to LHb neurons via GPi (Hong et al., 2019; Hong and Hikosaka, 2013). Thereby, this input signal from the dorsal striatum and GPi has been thought to be a critical source of phasic RPE response projecting into LHb and DA neurons.

**Figure 7:**
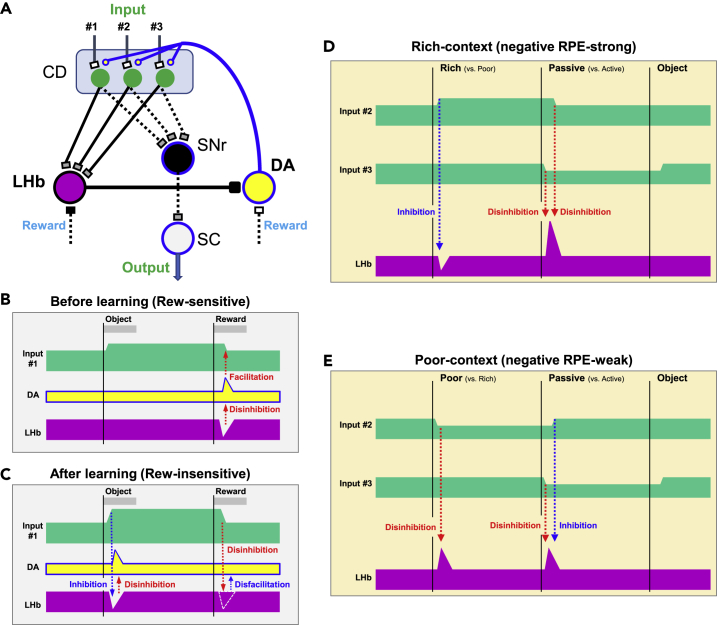
Hypothetical anticipatory-RPE circuits selectively modulated by previous-RP and updated-RP (A) Multiple inputs and a feedback loop of LHb with DA neurons via caudate nucleus. (B and C) Hypothetical phasic and tonic LHb input signals before (B) and after (C) learning of reward object. (D and E) Hypothetical tonic LHb input signals in two sequential reward events.

It was discovered that DA neurons are important for goal-directed behavior to obtain rewards (Schultz, 1998). DA neurons are activated by a reward outcome and then modulate the synaptic input to their target areas, especially the caudate nucleus (CD) (Kim et al., 2015; Tsutsui-Kimura et al., 2020) (Figure 7A). Then, CD can facilitate the output by two sequential inhibitions (i.e., disinhibition) through the substantia nigra reticulata (SNr) and the superior colliculus (SC) (Hikosaka et al., 2019), which is relevant to reward-directed behavior. However, this mechanism would occur after the reward, which may be too late to control goal-directed behavior. Instead, it is important to activate the goal-directed behavior when the reward-predicting object appears.

This may occur through the interaction between DA, CD, and LHb (Figure 7A). During goal-directed behaviors, many neurons in the caudate head (CDh) are tonically active between two sequential events (e.g., object onset until reward, Figures 7B and 7C) (Hikosaka et al., 1989). When reward is given, LHb neurons are inhibited and then DA neurons are excited, which would strengthen the input to CD neurons that are controlled by the input from DA neurons (Figure 7B). Then, the tonic activity between the object onset and the reward outcome would be increased (Figure 7C) (Kawagoe et al., 1998; Lauwereyns et al., 2002). This would be caused by a particular input (e.g., Input #1).

Then, LHb neurons would receive the tonic excitatory input from CD neurons, but show phasic responses because the input is mediated mainly by GPi neurons which are phasically active (Hong and Hikosaka, 2008): 1) phasic inhibition by object onset (by inhibition from CD) (Figure 7C, left), 2) loss of inhibition by reward (by disinhibition from CD) (Figure 7C, right). These data will cause the change of DA neuron activity (Figure 7C, DA): 1) phasic excitation by objects (by disinhibition from LHb), 2) loss of excitation by reward (by continued inhibition from LHb). These data would explain the Anticipatory-RPE in LHb as well as DA neurons.

Similar effects would occur during multiple sequential tasks (Figures 7D and 7E) which we tested in our experiments (Figure 1). For example, another group of CD neurons would be sensitive to the stage from environment (Rich versus Poor) until the stage of action (Active versus Passive) which would be caused by another input (e.g., Input #2): 1) tonic excitation by Rich environment (Figure 7D, left), 2) tonic inhibition by Poor environment (Figure 7E, left). These data are equivalent to Figures 1 and S1. Each of these tonic activities caused phasic changes in LHb neurons: 1) phasic inhibition by Rich environment (Figure 7D, left), 2) phasic disinhibition (excitation) by Poor environment (Figure 7E, left). These theoretical data are relevant to the experimental data in Figure 3E.

We then checked LHb activity under the same condition (i.e., passive) (Figures 7D and 7E, center) which would be caused by another input (e.g., Input #3). Because passive condition causes tonic inhibition in CD neurons (as suggested in Figure S1, right), it causes a phasic excitation (by disinhibition) in LHb neurons (Figures 7D-center and 7E-center). However, the preceding condition caused different effects on LHb neurons through Input #2: 1) the end of tonic excitation in Rich environment causes the decrease of inhibition (disinhibition = excitation) (Figure 7D, center), 2) the end of tonic inhibition in Poor environment causes the increase of inhibition (inhibition) (Figure 7E, center). These data together cause the difference in phasic activity: 1) strong excitation after Rich condition, 2) weak excitation after Poor condition. This actually occurred in our data (Figure 4K, especially at the beginning part).

These theoretical and experimental data suggest that LHb-DA mechanism can evaluate the significance of each step of the sequential processes toward the final outcome value, which should be based on long-term learning and memories. This is important because the subject can evaluate the significance of each step or action among many steps, which would be significant in real life.

### Implications of Anticipatory-RPE in long-term and short-term memories: From goal-directed Behavior to habit formation

How does reward information guide instrumental behaviors of animals? The information-processing has been globally studied in long-term and short-term memories. How then are these different sets of memories used together in the behaviors after learning? We suggest that LHb neurons may process both long-term and short-term memories of reward information. Recent studies found that reward information in short-term flexible value and long-term stable value memories are respectively processed in caudate head (CDh)- and caudate tail (CDt)-circuits in basal ganglia (Kim et al., 2015; Yasuda and Hikosaka, 2015). Here CDh and CDt receive signals from DA neurons in the rostral-medial part and caudal-lateral part of substantia nigra pars compacta (SNc) in parallel. A recent rodent study found that the anterior striatum and tail of the striatum signal different sensitivity of dopamine responses between the outcome and reward predictive cue (Tsutsui-Kimura et al., 2022). These are consistent with the findings in human brain (Balleine and O'Doherty, 2010) that are sensitive to the learning of outcome-response association (i.e., goal-directed behavior) in the anterior striatum (Haber et al., 2006; Ongur and Price, 2000) and sensitive to the learning of stimulus-response association (i.e., habitual behavior) in posterior lateral striatum (Tricomi et al., 2009).

A previous study found that LHb firmly controls the DA neurons in the rostral-medial part of SNc (Matsumoto and Hikosaka, 2007). In this previous study, LHb neurons were sensitive to the flexibly changed position-reward contingency and the changes in the LHb response were quicker than changes in the behavior according to the position-reward contingency. This suggests the LHb neurons are sensitive to the learning of the outcome-response association for the goal-directed behavior. However, in addition to the outcome-response association, our present study found that the LHb neurons are sensitive to the Anticipatory-RPE that is induced by the stimulus-response associations before the outcomes. This implies that LHb could play a crucial role in bridging the gap between the long-term experience of goal-directed behaviors and habit formation. The LHb is a distinguished brain area that can modulate both monoamine cell groups, DA and serotonin, in the SNc and dorsal raphe nucleus (DRN) (Herkenham and Nauta, 1979). The serotonin neurons in DRN have been suggested as one of the inputs to tail of striatum (Griggs et al., 2017; Jiang and Kim, 2018). Moreover, DRN projects to DA neurons in SNc (Watabe-Uchida et al., 2012) which can be an important candidate to innervate long-term and short-term reward signals carried in SNc.

Animal foraging behavior is often performed using both long-term and short-term memories. Long-term memory is useful for executing an automatic behavioral pattern to maximize reward in the long run. Meanwhile, in the series of action sequences, the pattern includes flexible behavioral adjustment depending on each uncertain reward event using short-term memory. In the present task, LHb neurons represented both long-term and short-term memories, which were switched quickly. For example, first, LHb responded to a long-termly experienced reward. At each step, LHb responded to contextual Anticipatory-RPE based on the average reward amount experienced by many different trials and blocks (Figures 3 and 4). In this task, each contextual event (environment, action cue) could be followed by a completely different outcome. Here, LHb neurons showed memories of many visual stimuli, including 16 environments (4 per context) and 80 objects (5 per environment) per set. Then, LHb represented RP based on the average of many experiences that requires long-term memory with high capacity.

On the other hand, LHb responded to short-term RP related to immediate action. In the active mode, good and bad objects had the same long-term RP in each environment context (Figures 5A–5D, orange line) as monkeys could obtain reward after skillful instrumental actions whatever appeared. There was only different short-term RP for immediate action based on the appearance-rate of the object (Figures 5A–5D, black dotted line). The short-term RP was temporarily reduced than long-term RP before object onset so that LHb neurons could react to good and bad objects differently.

However, because these object-based changes in neuronal activity were temporary, after completing appropriate required actions, LHb neurons continued to be sensitive to long-termly experienced reward contexts. At the end of the trial, LHb showed an absence of reward response to the long-termly experienced and certainly predicted reward outcome sequence (Figures 2A and 2C). These data suggest that the LHb neurons are sensitive to both long-term environmental contexts (Figures 3 and 4) and short-term goal-directed objects (Figure 5). The reward processing between long-term and short-term conditions in LHb could play crucial roles in the resilience and adaptability/flexibility functions of the emotional brain.

### Limitations of the study

We found that the LHb neurons encoded a step-by-step RPE signal at each event in multiple steps by a sequential presentation of distinct environments and objects. This finding could be important to develop therapeutic approaches in Parkinson disease patients who have disturbances of sequential movements through further studies on the LHb-DA network. For this reason, it will be important in the future to study the broad input and output of LHb neurons, including brain stem areas executing the actual motor function of animals.

## STAR★Methods

### Key resources table

**Table:** 

REAGENT or RESOURCE	SOURCE	IDENTIFIER
**Software and algorithms**		

Blip	Hong et al.	http://www.robilis.com/blip/
Prism8	GraphPad	https://www.graphpad.com/scientific-software/prism/
MATLAB	MathWorks	https://www.mathworks.com/RRID:SCR_001622

**Other**		

Electrode	Alpha-Omega	Cat#380-130610-00
EyeLink 1000 Plus	SR Research	RRID:SCR_009602
Micro-manipulator	Narishige	N/A

### Resource availability

#### Lead contact

Further information and requests for resources and reagents should be directed to and will be fulfilled by the lead contact, Hyunchan Lee (dr.hyunchan@gmail.com).

#### Materials availability

This study did not generate new unique reagents.

### Experimental model and subject details

Two adult male rhesus monkeys, CH and KI (8-year-old, 10–12 kg), were used for this study. All animal care and experiment procedures were approved by Animal Care and Use Committee of the National Eye Institute and complied with the Public Health Service Policy on the Humane Care and Use of Laboratory Animals. The monkey sat in a chair facing a screen in an electrically shielded and sound attenuated darkened room. Visual stimuli were rear-projected on the screen by a digital light processing projector (PJD8353s, ViewSonic). The eye position of the monkey was sampled at 1 kHz using a video-based eye tracker (EyeLink 1000 Plus, SR Research). Apple juice was used as a reward for the monkeys through a spout placed in front of their mouth. Apple juice was provided for 300 ms (about 600 μl) as a high-valued reward in the Rich-contexts, whereas low-valued rewards in the Poor-contexts opened the spout for 100 ms (about 200 μl). Airpuffs (10–20 psi) were used as punishment delivered for 100 ms through a tube placed 10 cm from the face. All the stimuli of the behavioral task, spike isolation, data acquisition, and data monitoring were controlled by the visual C++ software Blip (www.robilis.com/blip/).

### Method details

#### Multi-stage contingency task

##### Environment

In the task procedure, monkeys experienced environments, action cues, and objects in sequence. There were pseudo-randomized trials (416 trials/block) that started with the appearance of an environment (384 trials, 96 trials × 4 contexts) at the center of the screen or delivery of an uncued free outcome (free reward, 16 trials; free airpuff, 16 trials). The appearance of the environment (total 16 images/set; 4 images/context/set) establishes one of the four contexts (Rich-Safe, Poor-Safe, Rich-Dangerous, Poor-Dangerous) depending on reward valence (outcome type: reward or airpuff, contingency, and size) in following active and passive modes (Figure 1). In the Rich-contexts, monkeys experienced bigger rewards than Poor-contexts. Dangerous-contexts contained punishment in the passive mode, whereas Safe-contexts contained the same amount of reward in the active mode of those contexts. The environments were large (circular, diameter: 40°) grayscale landscape and face images from Google Earth (https://www.google.com/earth) and OpenAerialMap (https://openaerialmap.org) that were used in previous studies (Kunimatsu et al., 2019; Maeda et al., 2018) and Face Database (https://fei.edu.br/∼cet/facedatabase.html). The neuronal and behavioral data in the face environment were published in the related paper (Lee and Hikosaka, 2022). Monkeys experienced 2 sets of stimuli (total 32 environments and 160 fractal objects) in separated blocks. All trials were presented with 7 s intertrial intervals.

##### Active mode

The active mode was modified from the foraging task (Maeda et al., 2018) and the sequential saccade choice task (Amita and Hikosaka, 2019) in the previous studies. After a delay of 1 s from environment onset, an action cue (2° × 2°) among active-action and passive-action cues appeared at the environment’s center. The environment is sustained at the background of further visual stimuli until the end of each trial. The shape of the action cue reflected the task type of the trial. Active-action cue (magenta square) requires the monkey to hold gaze on the cue for 700 ms (192 trials, 48 trials × 4 contexts). After the fixation, good (33.3% earlier than bad object) or bad object appeared pseudorandomly at left or right position (15° from the center). Respective environments contained two different fractal images (total 32 images/set, 2 fractals × 16 environments/set) as good and bad objects. The objects were created by fractal geometry (∼10° × 10°) (Yamamoto et al., 2012). Correct fixation (>500 ms) to the good object provides a reward that was associated with the object (objects of Rich-contexts: high-valued reward, objects of Poor-contexts: low-valued reward). If monkeys made a fixation on the bad object, the trial was terminated with an error tone and disappearance of all stimuli. The trial was repeated from environment onset after a timeout (3 s). Failed saccade or broken fixation on the active-action cue or good object also terminated the trial and restarted the trial after the timeout. After correct avoidance (fixation <500 ms or neglection for 1 s), the bad object disappeared, and the active-action cue re-appeared. After the fixation to the re-appeared action cue, the good object appeared and provided a reward after the correct fixation.

##### Passive mode

The passive mode was modified from the Pavlovian task in the previous study (Matsumoto and Hikosaka, 2009a). After 1 s from environment onset, a passive-action cue (magenta circle) appeared (192 trials, 48 trials × 4 contexts). The passive-action cue disappeared after another 1 s and an object appeared without any action requirement. Each environment contained three respective objects (total 48 images/set, 3 fractals × 16 environments/set) for the passive mode which were associated with 100%, 50%, and 0% probabilities of reward or airpuff. Objects in the Safe-contexts were associated with reward (objects of Rich-contexts: high-valued reward, objects of Poor-contexts: low-valued reward). In the Dangerous-contexts, objects were associated with airpuff. The objects were presented with pseudorandom (100%, 16 trials/context; 50%, 16 trials/context; 0%, 16 trials/context). The outcome was delivered 1.5 s after the object onset.

#### **Single unit recording**

The single neurons in LHb were recorded by a plastic recording chamber which was implanted over the midline and tilted posteriorly by 8°. The recording sites were determined by MRI scanning (4.7 T, Bruker) with a gadolinium-filled 1 mm spacing grid system. In the scanned MR images, recording sites were detected by electrode path from the grid hole inside of the recording chamber. LHb neurons were searched around +7 mm to interaural based on the stereotaxic atlas (Saleem and Logothetis, 2012). We recorded and analyzed 33 LHb neurons in two monkeys (15 in monkey CH and 18 in monkey KI). In this experiment, we focused on the presumed LHb neurons that were sensitive to unpredicted reward delivery, omission, and punishment with phasic inhibition and excitation (Figures 2 and S2). LHb neurons were recorded by a glass-coated electrode (diameter 0.38 mm, 1 MΩ, Alpha-Omega) that was advanced through a guide tube by an oil-driven micro-manipulator (MO-97A, Narishige). The neuronal signals were amplified at a gain of 10k and filtered from 0.1 to 10 kHz by a microelectrode AC amplifier (model 1800; A-M Systems) and a band-pass filter (model 3384; Krohn-Hite). The single neurons were isolated online and collected at 1 kHz using a custom voltage- and time-based window discriminator in the software Blip.

### Quantification and statistical analysis

Data were presented as mean ± standard error of the mean (SEM) and analyzed using MATLAB (MathWorks) and Prism8 (GraphPad Software). Firing rates were smoothed with a Gaussian kernel (σ = 10 ms). The mean baseline firing rate of each neuron was calculated during 1000 ms before the environment onset. The statistical significances of the neuronal and behavioral discriminations were tested using one-way analysis of variance (ANOVA) and two-way repeated measures ANOVA with Tukey posthoc test. The correlations between neuronal and behavioral responses were analyzed by Pearson correlation analysis.

## Data Availability

• All data reported in this paper will be shared by the lead contact upon request. • This paper does not report original code. • Any additional information required to reanalyze the data reported in this paper is available from the lead contact upon request.
