# Supplementary material for: Lateral habenula neurons signal step-by-step changes of reward prediction
Source: iScience. 2022 Oct 27;25(11):105440. doi: 10.1016/j.isci.2022.105440 (PMC9641246; doi:10.1016/j.isci.2022.105440)
Supplement: Document S1. Figures S1–S7 [file mmc1.pdf]

iScience, Volume *25*

Supplemental information

Lateral habenula neurons signal step-by-step changes of reward prediction

Hyunchan Lee and Okihide Hikosaka

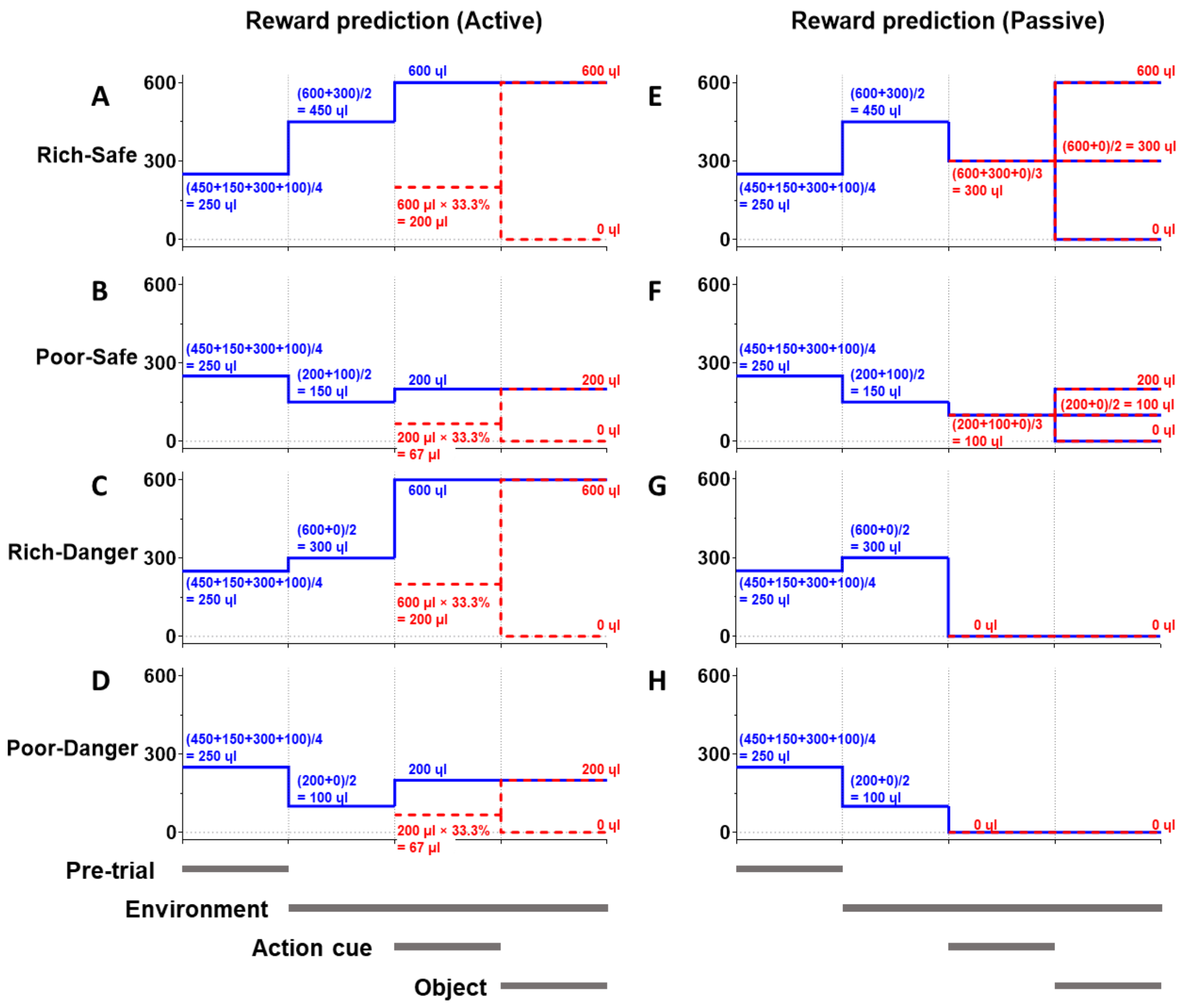

Figure S1. Theoretical Reward and Punishment Predictions during Task Procedure, Related to Figure 2

(A-D) Theoretical reward predictions during active mode. Theoretical reward predictions were determined by the combinations of task conditions, environment and action contexts (blue) and appearance-rate (red dotted) of objects (good object, 33.3%). (E-H) Theoretical reward predictions during the passive mode.

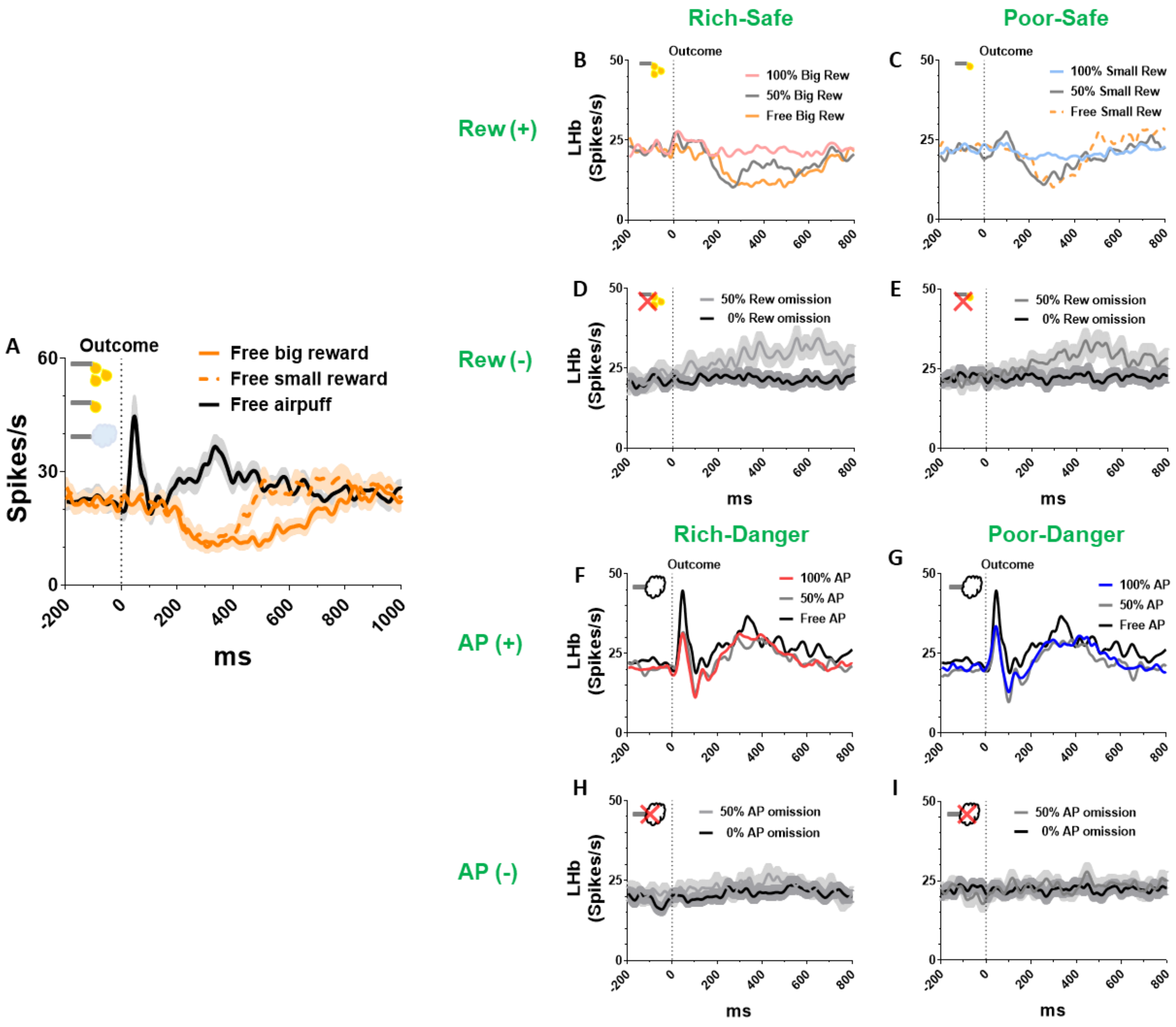

Figure S2. LHb Responses to Free Outcome, Related to Figure 2

(A) Population activity of LHb neurons in response to the uncued free high/low-valued reward and punishment. LHb neurons were excited by punishment and inhibited by reward. Reward response was more inhibited by high-valued reward than low-valued reward. (B, C, F, G) The comparison of population activities of LHb neurons in response to the cued 100% and 50% outcomes in the passive mode and the uncued free high/low-valued reward and punishment (shown in A). (D, E, H, I) Population activity of LHb neurons in response to the 50% and 0% cued high/low-valued reward and punishment omission.

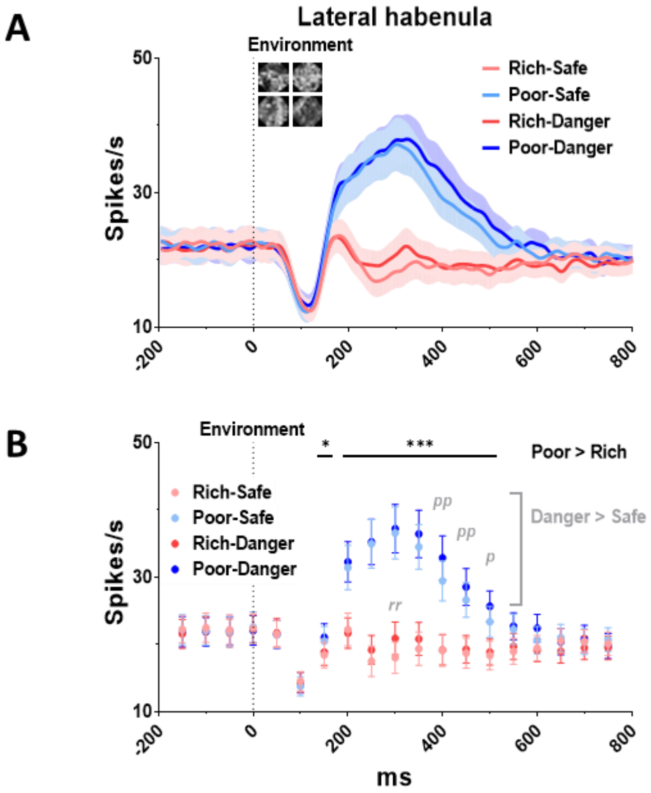

Figure S3. Statistical Analysis of LHb Responses to Environment, Related to Figure 3
(A) Population activity of LHb neurons in response to four environments (shown in Fig. 3E). (B) Same as (A), but the activity is shown as a temporal sequence (50 ms bin). In each time bin, the difference of the population activity was analyzed statistically in each dimension of environment: Poor > Rich ($P < 0.001$, two-way repeated measures ANOVA; $^{*}P < 0.05$, $^{***}P < 0.001$, Both Rich-Safe vs. Poor-Safe and Rich-Dangerous vs. Poor-Dangerous, Tukey posthoc test); Dangerous > Safe ($^{rr}P < 0.01$, Rich-Safe vs. Rich-Dangerous; $^{p}P < 0.05$, $^{pp}P < 0.01$, Poor-Safe vs. Poor-Dangerous, Tukey posthoc test).

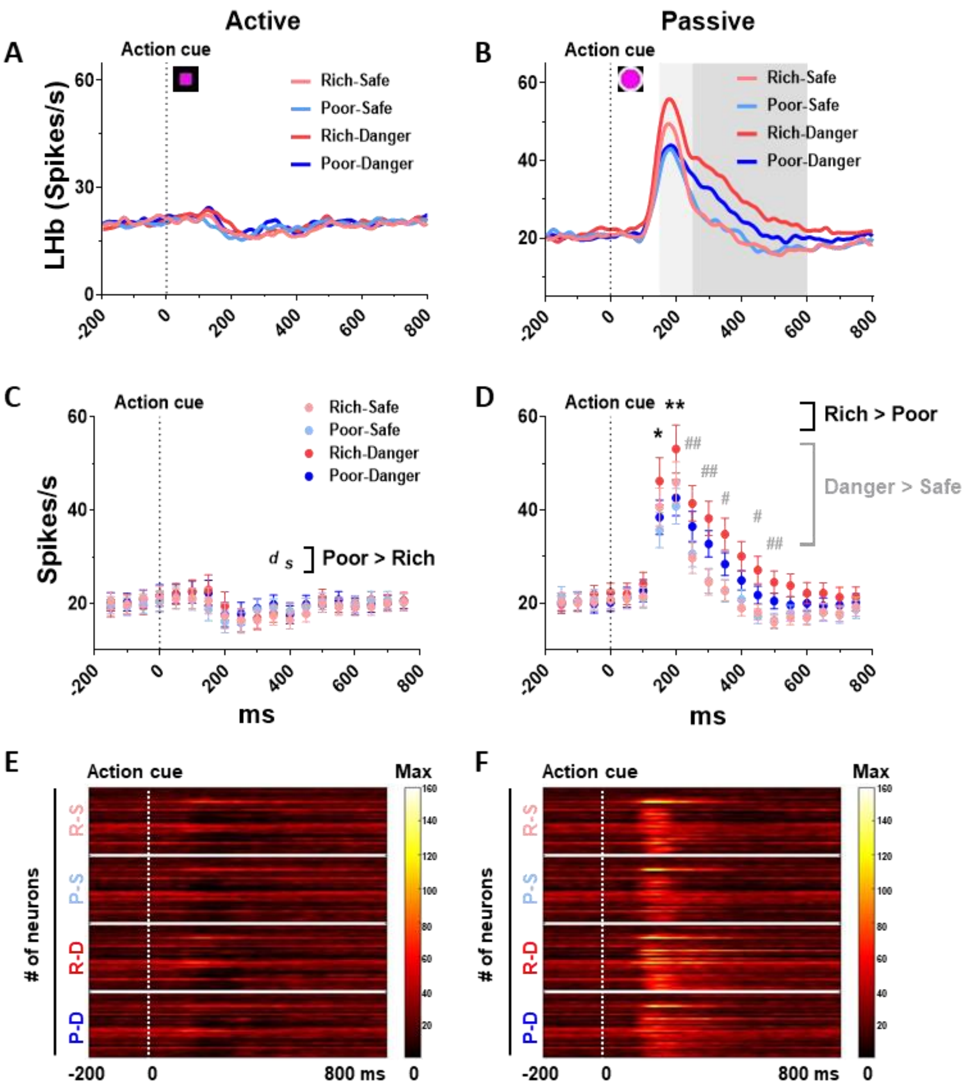

Figure S4. Statistical Analysis of LHb Responses to Action Cue, Related to Figure 4

(A-B) Population activity of LHb neurons in responses to active- and passive-action cues (shown in Fig. 4I and K). (C-D) Same as (A-B), shown as a temporal sequence (50 ms bin). The population activities in each time bin were statistically analyzed in each dimension of environment: Poor > Rich ($P < 0.05$, two-way repeated measures ANOVA; $^{s}P < 0.05$, Rich-Safe vs. Poor-Safe; $^{d}P < 0.05$, Rich-Dangerous vs. Poor-Dangerous, Tukey posthoc test); Rich > Poor ($P < 0.001$, two-way repeated measures ANOVA; $^{*}P < 0.05$, $^{**}P < 0.01$, Both Rich-Safe vs. Poor-Safe and Rich-Dangerous vs. Poor-Dangerous, Tukey posthoc test); Dangerous > Safe ($^{\#}P < 0.05$, $^{\#\#}P < 0.01$, Both Rich-Safe vs. Rich-Dangerous and Poor-Safe vs. Poor-Dangerous, Tukey posthoc test). (E-F) The responses of each LHb neuron.

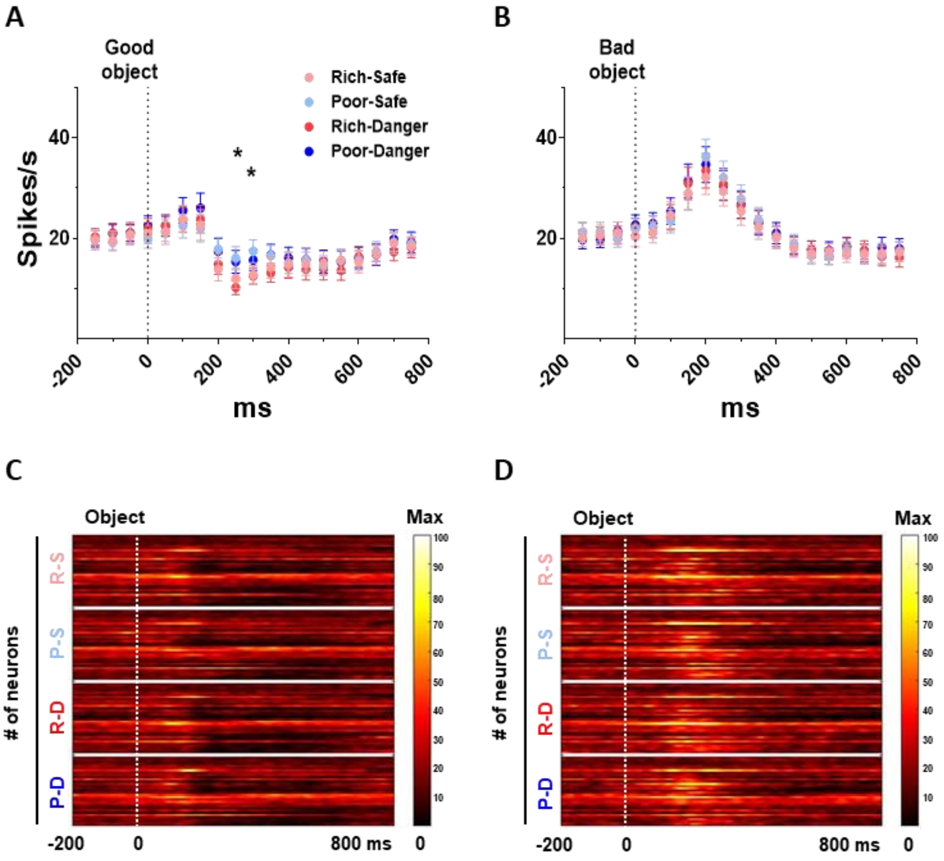

Figure S5. Statistical Analysis of LHb Responses to Objects in Active Mode, Related to Figure 5

(A) Population activity of LHb neurons in responses to good object. LHb was more suppressed in Rich-contexts than Poor-contexts ($P < 0.05$, two-way repeated measures ANOVA; *$P < 0.05$, Both Rich-Safe vs. Poor-Safe and Rich-Dangerous vs. Poor-Dangerous, Tukey posthoc test). (B) Averaged response of LHb to bad object. There was an insignificant difference between contexts. (C-D) The responses of each LHb neuron.

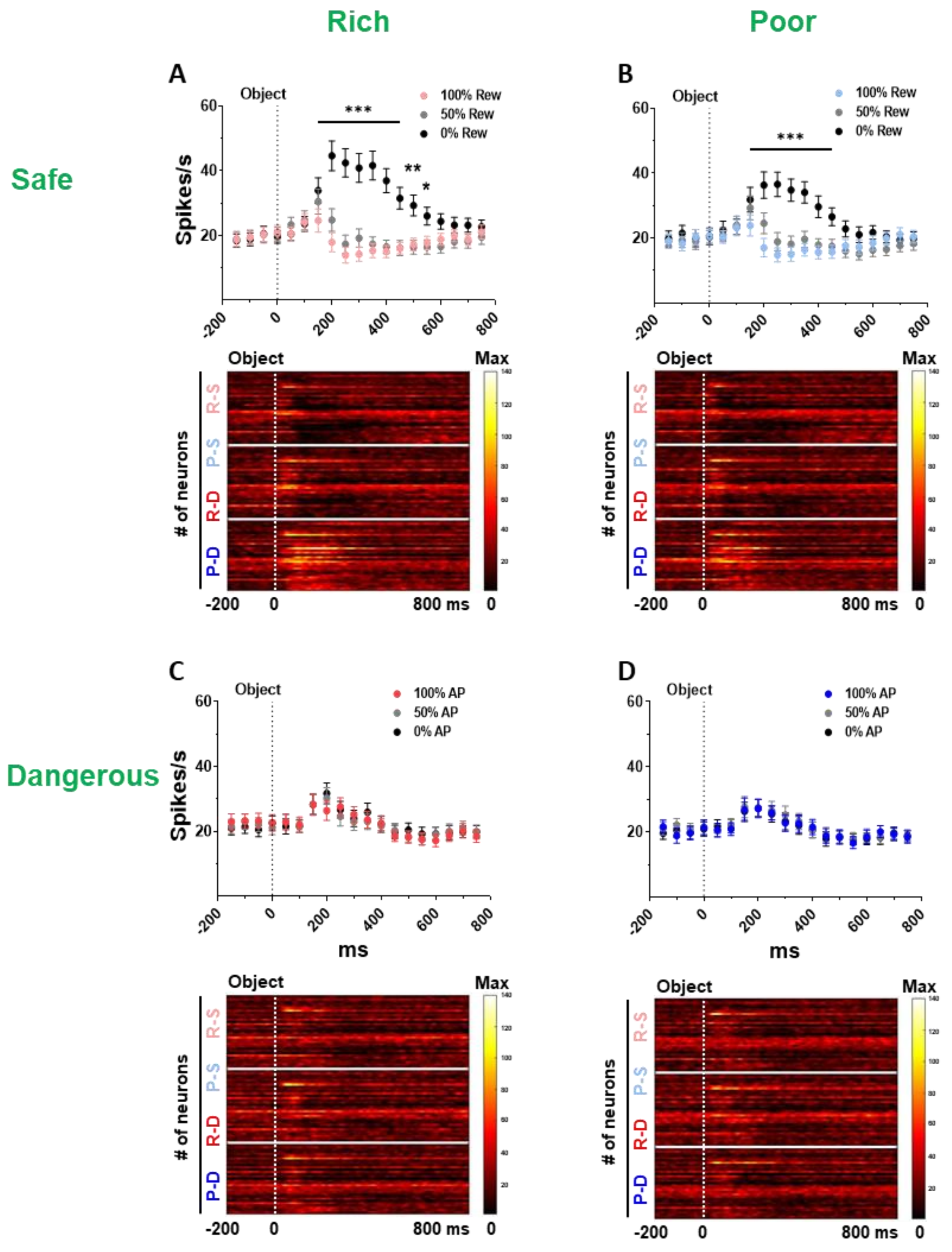

Figure S6. Statistical Analysis of LHb Responses to Objects in Passive Mode, Related to Figure 6

(A) Population activity of LHb neurons in responses to CS in Rich-Safe context. LHb was more suppressed by 100% and 50% CS than 0% CS (*P* < 0.001, two-way repeated measures ANOVA; **P* < 0.05, ***P* < 0.01, ****P* < 0.001, 100% CS vs. 0% CS, Tukey posthoc test). (B) Population activity of LHb neurons in responses to CS in Poor-Safe context. LHb was more suppressed by 100% and 50% CS than 0% CS (*P* < 0.001, two-way repeated measures ANOVA; ****P* < 0.001, 100% CS vs. 0% CS, Tukey posthoc test). (C) Population activity of LHb neurons in responses to CS in Rich-Dangerous context. (D) Population activity of LHb neurons in responses to CS in Poor-Dangerous context. There were insignificant differences between LHb responses to 100%, 50%, 0% CS in Dangerous-contexts. (A-D) Bottom, the responses of each LHb neuron.

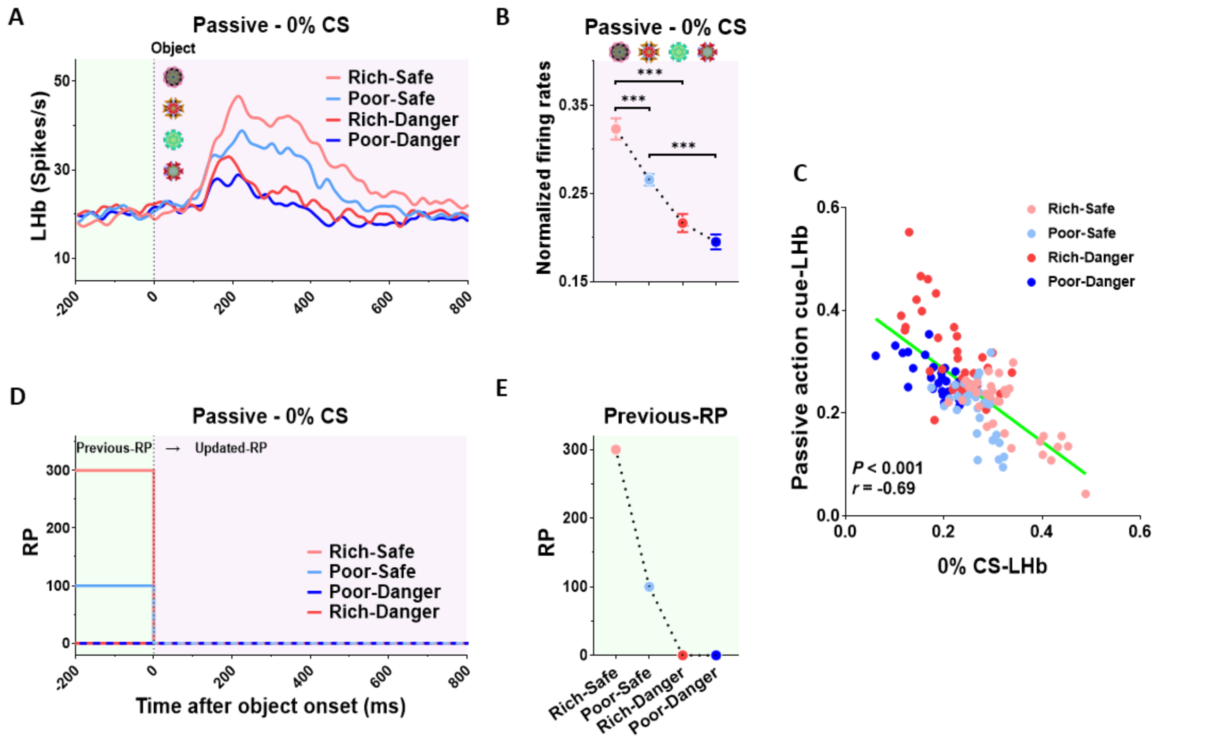

Figure S7. The Effect of Preceding Reward Prediction in LHb Responses to 0%-Reward Object, Related to Figure 6

(A) Population activity of LHb neurons in responses to 0% CS in passive mode. (B) Normalized LHb response to 0% CS during 150-600 ms after the object onset. Lateral habenula neurons were excited by 0% CS stronger in Safe-contexts than Dangerous-contexts ($P < 0.001$, one-way ANOVA; ***$P < 0.001$, Tukey posthoc test). Moreover, the neuronal responses in the Safe-contexts were more increased in the Rich-context than Poor-context. (C) Correlations of normalized LHb responses between passive-action cue (step 2) and continuous neuronal response to 0% CS (step 3). LHb response to 0% CS was significantly correlated with the response to passive-action cue (late phase, 250-600 ms) in preceding step ($r = -0.69$, P < 0.001). (D) Theoretical RP to 0% CS. (E) Previous-RP before 0% CS onset. Theoretical RP was higher in Safe-contexts than Dangerous-contexts before the 0% CS onset (Previous-RP, Rich-Safe, 300; Poor-Safe, 100; Rich-Dangerous, 0; Poor-Dangerous, 0).
